# β-Boomerang Antimicrobial and Antiendotoxic Peptides: Lipidation and Disulfide Bond Effects on Activity and Structure

**DOI:** 10.3390/ph7040482

**Published:** 2014-04-21

**Authors:** Harini Mohanram, Surajit Bhattacharjya

**Affiliations:** School of Biological Sciences, Nanyang Technological University, 60 Nanyang Drive, Singapore 637551, Singapore; E-Mail: M.HariniBalaji@ntu.edu.sg

**Keywords:** antimicrobial peptides, NMR, β-boomerang peptide, antibiotics, endotoxins, LPS

## Abstract

Drug-resistant Gram-negative bacterial pathogens and endotoxin- or lipopolysaccharide (LPS)-mediated inflammations are among some of the most prominent health issues globally. Antimicrobial peptides (AMPs) are eminent molecules that can kill drug-resistant strains and neutralize LPS toxicity. LPS, the outer layer of the outer membrane of Gram-negative bacteria safeguards cell integrity against hydrophobic compounds, including antibiotics and AMPs. Apart from maintaining structural integrity, LPS, when released into the blood stream, also induces inflammatory pathways leading to septic shock. In previous works, we have reported the *de novo* design of a set of 12-amino acid long cationic/hydrophobic peptides for LPS binding and activity. These peptides adopt β-boomerang like conformations in complex with LPS. Structure-activity studies demonstrated some critical features of the β-boomerang scaffold that may be utilized for the further development of potent analogs. In this work, β-boomerang lipopeptides were designed and structure-activity correlation studies were carried out. These lipopeptides were homo-dimerized through a disulfide bridge to stabilize conformations and for improved activity. The designed peptides exhibited potent antibacterial activity and efficiently neutralized LPS toxicity under *in vitro* assays. NMR structure of C4YI13C in aqueous solution demonstrated the conserved folding of the lipopeptide with a boomerang aromatic lock stabilized with disulfide bond at the C-terminus and acylation at the N-terminus. These lipo-peptides displaying bacterial sterilization and low hemolytic activity may be useful for future applications as antimicrobial and antiendotoxin molecules.

## 1. Introduction

Development of novel antimicrobial peptides (AMPs) is urgently needed to combat against rapidly appearing drug resistant strains [1,2,3]. AMPs are generally short in length and composed of relative proportions of cationic and hydrophobic residues [3,4,5,6]. AMPs may be categorized based on their structures namely (a) α-helix, (b) β-sheet or β-hairpin stabilized by disulfide bridges, (c) extended and (d) loops with or without disulfide bonds. The cytolytic activity of most AMPs stems from membrane- targeted non-receptor mediated mechanisms and hence development of drug resistance towards such peptides would be difficult [7,8,9,10,11]. The structurally divergent nature and simple mode of action of AMPs make them suitable candidates for the development of anti-infective agents [12,13]. Lipopolysaccharide (LPS) of the outer membrane may restrict activity of AMPs to Gram-negative bacteria [14,15,16,17,18]. As a mode of action, broad spectrum AMPs would disrupt the permeability barrier of LPS-outer membrane, which is an important step for killing bacterial cells [18,19]. In addition, LPS or endotoxin induces inflammatory pathways leading to the production of cytokines that often results in septic shock [19,20,21]. Sepsis or septicemia occupies the tenth position among the most dreadful diseases and there are no recent significant improvements for the treatment of sepsis-affected patients [22,23,24].

Among the various classes of antimicrobial peptides, β-hairpins stabilized by disulfide bridges, including defensins, protegrins, polyphemusins and tachyplesins, occupy a prominent position [25,26,27]. The disulfide bridges are found to play an important role in the antibacterial activity and cell selectivity [28,29,30]. Moreover, disulfide bridges can stabilize the folded conformations of AMPs in aqueous solutions that upon membrane interactions may form higher order structures [31]. Addition of fatty acyl chains to AMPs shows increased microbial sterilization probably by increasing incorporation of peptides deeply into the membrane [32,33,34,35,36,37,38,39,40]. We are interested in developing AMPs that disrupt the LPS outer membrane as a mode of action [41,42,43,44,45,46]. In previous reports, we have designed a set of short (~12 amino acid long) cationic/hydrophobic peptides with antibacterial and antiendotoxic activities [41,42]. The designed peptides have been termed β-boomerangs as they adopt boomerang-like structures in complex with LPS. From an initial design, the second generation of β-boomerang peptides demonstrated enhanced LPS neutralization and antibacterial activity. Solution NMR studies revealed packing of two aromatic residues (W4 and F9) and amphipathic segregation of four cationic residues is critical for antimicrobial and antiendotoxic activities [42]. In this work, we have utilized the most active analog of the second generation of β-boomerang peptide, YI12WF (YVLWKRKRFIFI-amide) as a starting template for further design of potent antimicrobial activity and LPS neutralization peptide analogs. Toward this end, we have prepared analogs of YI12WF containing five basic residues (YVLW*KRKRK*FCFI-amide) in the loop of the β-boomerang structure and which are acylated at the N-termini, by C4 and C8 compounds. Further, residue I11 has been replaced by residue Cys to prepare disulfide linked variants of β-boomerang lipopeptides. All of the peptides are tested for antimicrobial and LPS neutralization and RBC lysis. Biophysical studies, using optical spectroscopic methods, ITC and dynamic light scattering have provided molecular insights into the mode of action of the designed peptides in Gram-negative bacterial outer membrane and endotoxin neutralization. Atomic-resolution structure, by NMR spectroscopy, has been determined for one of the potent peptides. Collectively, current results describe structure and activity of highly potent β-boomerang peptides of superior activity in comparison to parent peptides. These β-boomerang lipopeptides may be used in developing non-toxic antibacterial therapeutics.

## 2. Experimental Section

*Materials*: LPS of *E. coli* 0111:B4, FITC-LPS of *E. coli* 055:B5, acrylamide, NPN (1-*N*-phenylnaphthylamine) were purchased from Sigma (Saint Louis, MO, USA). All bacterial strains were obtained from the American Type Culture Collection (Manassas, VA, USA). Designed peptides were synthesized commercially by GL Biochem (Shanghai, China) and further purified by a reverse phase HPLC (Waters), using a C_18_ column (300 Å pore size, 5 μm particle size). The dimeric variants of β-boomerang lipopeptides were treated with 20% DMSO for 18 hours and the oxidized peptides were further purified by HPLC. A linear gradient of acetonitrile/water mixture was used for the purification and fractions eluting with higher purity were collected and freeze-dried.

*Antibacterial assay*: The minimum inhibitory concentration (MIC) of the designed peptides was determined against four Gram-negative strains (*E. coli*, *P. aeruginosa* ATCC 27853, *K. pneumoniae* ATCC 13883, *S. enterica* ATCC 14028) and four Gram-positive strains (*B. subtilis*, *S. aureus* ATCC 25923, *S. pyogenes* ATCC 19615 and *E. faecalis* ATCC 29212). In brief, mid logarithmic phase of overnight grown bacterial cultures were obtained in LB broth and adjusted to OD_600_ 0.2 in 10 mM phosphate buffer, pH 6.8. About 50 μL of these cells were added to equal volume of designed peptides at two-fold dilutions in 96-well sterile polypropylene plates followed by incubation for 3hrs at 37 °C. Aliquots of the above titrated wells were streaked onto Mueller-Hinton agar plates and the concentration at which no visible bacterial growth was observed considered as the MIC value of the peptide.

*Hemolytic assay*: Blood was collected from healthy mice in a tube containing EDTA. Cells with EDTA were centrifuged at 800 g for 10 min, to remove the buffy plasma coat layer, and washed three times in PBS (35 mM phosphate buffer, 150 mM NaCl, pH 7.0). 50 μL of RBC solution was added to equal volume of two-fold dilution of the peptides in 96-well micro titer plates and incubated for one hour. The final erythrocyte concentration estimated to be ~4% (v/v). After one hour, the mixture was centrifuged and the release of hemoglobin in the supernatant was determined spectrophotometrically at OD_540_. Buffer and 1% Triton-X in the place of peptides used as negative control and positive control, respectively. The percentage of hemolysis was calculated using the following method:

Percentage of hemolysis = (OD peptide − OD buffer)/(OD Triton-X-OD buffer) × 100

*LPS neutralization assay*: LAL chromogenic kit (QCL 100 Cambrex, Walkersville, MD, USA) was used to determine endotoxin neutralization of the designed peptides following protocol of the vendor. LPS in Gram negative bacteria activates a proenzyme in limulus amoebocyte lysate (LAL). This activated enzyme in turn catalytically release a colored product pNA from the colorless substrate Ac-Ile-Glu-Ala-Arg-pNA which is detected spectrophotometrically at OD_410_. All peptides were dissolved in the pyrogen-free water supplied with the kit and pH was adjusted to 7.0 with 1N HCl or 1N NaOH (which is prepared in pyrogen-free water). The increasing concentrations of the peptides were incubated with three different concentrations of endotoxins, *i.e*., at 0.4, 0.8 and 1.0 endotoxin units (EU) in a total volume of 50 μL for 30 minutes at 37 °C. About 50 μL of LAL reagent was added to peptide-LPS complex and further incubated for 10 minutes followed by addition of 100 μL substrate. After incubation of six minutes for the reaction, the release of colored product was recorded at OD_410_. Water in the place of peptides served as negative control (blank) that is considered as 0% inhibition and percentage of LPS neutralization was calculated by:

% of LPS neutralization = [(OD blank − OD peptide)/OD blank] × 100

*Zeta potential measurements*: The zeta potential measurements were carried out on a Zetasizer Nano ZS (Malvern Instruments, Worcestershire, UK) equipped with a 633 nm He lasers. Mid-log phase grown bacterial cells were diluted to an OD_600_ of 0.2 and a basal measurement in the absence of peptides was acquired in disposable zeta cells with gold electrodes. Increasing concentrations of the designed peptides were then added to the cells and the measurements were made. A total of five measurements of 100 runs each were carried out for all dilutions.

*Intrinsic tryptophan fluorescence and acrylamide quenching*: The intrinsic Trp fluorescence spectra of the designed peptides were obtained by exciting samples at 280 nm and emission was collected from 300–400 nm in 10 mM sodium phosphate buffer. Fluorescence experiments were carried out with a Cary Eclipse (PaloAlto, CA, USA) fluorescence spectrophotometer using a 0.1 cm path length quartz cuvette. Experiments were initiated by obtaining emission spectra peptide (5 μM) alone followed by additions of increasing concentrations of LPS. Quenching experiments were conducted to examine the solvent accessibility of tryptophan residue in aqueous and hydrophobic environments. Acrylamide, a neutral quencher was added at increasing concentrations to free peptide and peptide/LPS complexes. The extent of decrease in fluorescence intensity was then used to calculate Stern-Volmer constant with the formula F_0_/F = 1 + K_sv_[Q]. F_0_ and F are the fluorescence intensities before and addition of acrylamide, K_sv_ is the Stern-Volmer constant and [Q] is the quencher concentration.

*Outer membrane permeability assay*: Outer membrane of Gram bacteria acts as permeability barrier for hydrophobic antibacterial compounds. Hence the ability of the designed peptides to permeabilize through the outer membrane was examined using 1-N-phenylnaphthylamine (NPN) dye. *E. coli* cells were grown to mid logarithmic phase and diluted to an OD_600nm_ of 0.5 in 10 mM sodium phosphate buffer, pH 7.0. The excitation was set at 350 nm and emission at 390–450 nm. NPN was added to the diluted cells at the concentration of 10 μM and a basal fluorescence was recorded followed by the addition of increasing concentration of peptides.

*Dissociation of FITC-LPS*: LPS molecules form micellar aggregates in water. The competency of the peptides to dissociate LPS micelles were studied using FITC conjugated LPS. In this method, blank fluorescence of 500 nM of FITC-LPS was recorded followed by the addition of increasing concentrations of peptides. The excitation was set at 480 nm and emission at 500–550 nm. 

*Dynamic light scattering measurements*: LPS forms micelles of large sizes, which with the addition of increasing concentration of peptides, dissociates to smaller sizes. DLS measurements were made for 0.5 μM LPS and then with LPS: Peptide ratios of 1:2 and 1:4. The scattering was measured with Dynamic Light Scattering software provided with the instrument (Brookhaven Instruments Corp., Holtsville, NY, USA) and the scattering data was analyzed with CONTIN method.

*Isothermal titration calorimetry*: Binding of designed peptides with LPS micelles was determined using isothermal titration calorimetry in VP-ITC micro calorimeter (Microcal Inc, Northampton, MA, USA). Peptides and LPS samples were dissolved in 10 mM phosphate buffer, pH 7.0 and filtered. LPS at a concentration of 10 μM was loaded into the sample cell and the reference cell was filled with the same buffer. The syringe was filled with 1mM peptide solution. Typically 30 injections of 3.5 μL of the peptides were made into the sample cell at 25 °C. Sample cell was stirred continuously at 300 rpm. Raw data were obtained and analyzed using single site binding model in Microcal Origin 5.0 software. Association constant (K_a_) and enthalpy change (ΔH) were directly determined from ITC profiles. Dissociation constant was calculated from K_d_ = 1/K_a._ ΔG and TΔS were estimated from the fundamental equations of thermodynamics, ΔG = −RTlnK_a_ and TΔS = (ΔH − ΔG), respectively.

*Structural characterization by NMR spectroscopy*: NMR spectra were recorded on a Bruker DRX 600 (Fallanden, Switzerland) spectrometer containing a cryo-probe and pulse field gradients. Data acquisition and processing were performed with the Topspin software (Bruker) running on a Linux workstation. Two dimensional TOCSY (total correlation spectroscopy) and NOESY (nuclear Overhauser effect spectroscopy) spectra of peptides in free solution were acquired in aqueous solutions containing 10% D_2_O at pH 4.5 with 0.5 mM peptide concentration. The mixing times were fixed at 80 ms for TOCSY and 250 ms for NOESY. 2,2-Dimethyl-2-silapentane 5-sulfonate sodium salt (DSS) was used as an internal chemical shift reference. Because of severe spectral overlaps at 298 K, spectra were acquired at low temperature of 278 K. NOESY experiments were performed with 456 increments in t_1_ and 2 K data points in t_2_ using the WATERGATE procedure for water signal suppression. NMR data processing and analysis were carried out using Topspin (Bruker) and Sparky (T.D. Goddard and D.G. Kneller, University of California, San Francisco, CA, USA) programs respectively. For tr-NOESY experiments, 0.5 mM peptide solutions were titrated with different concentrations of LPS from 5 μM to 50 μM. The two dimensional tr-NOESY NMR experiments were performed with the same parameters as NOESY experiment except with the mixing time of 150 ms. 

*NMR-derived structure calculations*: NMR structures were calculated using CYANA program [47]. Sequential walk of different spin systems were achieved in SPARKY by analyzing two dimensional TOCSY and NOESY spectra of the peptide either in free or in LPS complex. On the basis of cross peak intensities, NOEs were categorized to strong, medium and weak. They were then translated to upper bound distance limits to 2.5, 3.5 and 5.0 Å respectively. PROCHECK was used to analyze Ramachandran plot for the validation of structures calculated [48].

## 3. Results and Discussion

### 3.1. Peptide Design

**Figure 1:**
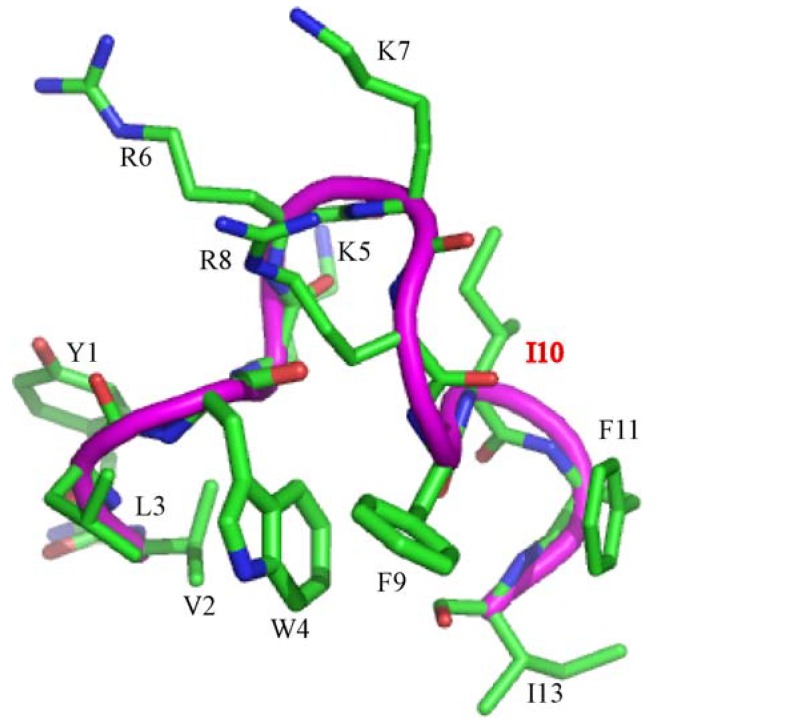
β-boomerang structure of YI12WF peptide. The close packing interactions between W4 and F9 are critical for the boomerang like fold of the peptide. Residue I10 has been highlited in red.

We have designed a set of short, 12-residue long, cationic/hydrophobic peptides with broad-spectrum antibacterial and antiendotoxic activities [41,42]. These peptides assume boomerang like structure in complex with LPS micelles and termed as β-boomerang peptides (Figure 1). The β-boomerang fold is defined by a long-range packing (i to i + 5) between two aromatic residues at positions 4 and 9 (Figure 1). Figure 1 shows the β-boomerang structure of a representative peptide YI12WF with the sequence Y-V-L-W-K-R-K-R-F-I-F-I-amide. SAR studies performed on the second generation of YI12 peptides have revealed that YI12WF peptide contains a relatively higher antimicrobial and antiendotoxic activities [42]. Here, we have utilized YI12WF as a template in the design of third generation of β-boomerang peptides for superior activity.

N-terminal acylation of antimicrobial peptides have been shown to possess improved bactericidal and antiendotoxic activity [34]. In addition, incorporation of disulfide bridges in AMPs may stabilize conformations and enhance cationicity and hydrophobicity required for enhanced activity. Towards these propositions, we have synthesized N-terminal acylated analogs, containing either C4 or C8 carbon chain, of YI12WF peptides and also Cys analogs, by replacing residue Ile10 to Cys, of acylated variants of YI12WF. Residue Ile10 was chosen for Cys replacement since this residue is located at the exposed face of the β-strand of the YI12WF structure (Figure 1). However, all of these analogs including acylated C4YI12WF and C8YI12WF, disulfide dimerized C4YI12WFC and C8YI12WFC remained insoluble in buffers and cannot be further characterized. In order to improve solubility, stability and cationicity a Lys residue has been introduced into the cationic loop of the YI12WF peptide, yielding YI13C peptide with sequence: Y-V-L-W-K-R-K-R-K-F-C-F-I-amide (Table 1). We have further prepared acylated analogs with C4 and C8 chains to YI13C (Table 1). In order to ascertain the role of aromatic Trp4 and Phe10 residues a peptide containing Ala residue (C8YI13CAA), replacing both Trp4/Phe10, has also been synthesized (Table 1). All of these designed peptides were purified with HPLC and the retention time of each peptide has been listed in Table 1. Overall, the acylated peptides were found to be more hydrophobic than non-acylated analogs as revealed by a comparatively higher retention time (Table 1).

**Table 1:** Amino acid sequences and HPLC retention times (R_t_) of of designed β-boomerang lipopeptide analogs.

Peptide designation	Primary structure	Retention time (R_t_)
YI13C	Y-V-L-W-K-R-K-R-K-F-C-F-I	27.48
C4YI13C	C4- Y-V-L-W-K-R-K-R-K-F-C-F-I	31.62
C8YI13C	C8- Y-V-L-W-K-R-K-R-K-F-C-F-I	37.40
C8YI13CAA	C8- Y-V-L-A-K-R-K-R-K-A-C-F-I	32.38

### 3.2. Antimicrobial Activity

The ability of the designed analogs to kill Gram-negative and Gram-positive bacteria were analyzed against four Gram-negative and four Gram-positive bacteria. MIC assays demonstrated that the incorporation of inter-molecular disulfide bond and acylation exerts strong bactericidal effects on all the Gram-negative and Gram-positive strains (Table 2).

**Table 2:** Minimum inhibitory concentration (MIC, in μM) of the designed peptides and hemolytic activity of the peptides.

Bacteria	YI13C	C4YI13C	C8YI13C	C8YI13CAA
**Gram-negative**				
*E.coli* (Lab strain)	12.5	10	3	50
*P.aeruginosa* (ATCC 27853)	20	15	5	100
*K. pneumoniae* (ATCC 13883)	25	8	12	100
*S.enterica* (ATCC 14028)	50	50	50	>200
**Gram-positive**				
*B.subtilis* (Lab strain)	20	15	5	50
*S.aureus* (ATCC 25923)	20	50	5	200
*S.pyogenes* (ATCC 19615)	50	50	50	>200
*E.faecalis* (ATCC 29212)	50	50	4	>200
% of hemolysis at 50 μM peptide concentration	21.5	14.1	21.5	30.2

The bactericidal activity of acylated analogs C4YI13C and C8YI13C, was found to be improved in comparison to non-acylated YI13C with C8YI13C showing the lowest MIC values (Table 2). It is noteworthy that the bactericidal activities of these peptides are somewhat strain-specific as higher lethal effects were observed against Gram-negative strains in comparison to Gram positive ones. Substitution of aromatic residues W, F, with Ala (C8YI13CAA) yielded rather inactive peptides (Table 2). The poor bactericidal activity of the C8YI13CAA may suggest that specific involvement of aromatic residues, Trp4 and Phe10, in determining activity of the β-boomerang peptides. Toxicity of our designed peptides was assayed using red blood cells. Overall, bactericidal peptides demonstrated low hemolytic activity estimated at a single concentration of 50 μM peptide (Table 2). Hemolysis seems to decrease with C4 acylation that slightly increases further with C8 acylation. The aromatic amino acid substitution (C8YI13CAA) analog has rendered to be more hemolytic indicating the potential specificity of aromatic packing (Table 2).

### 3.3. Neutralization of LPS by LAL Assay

**Figure 2:**
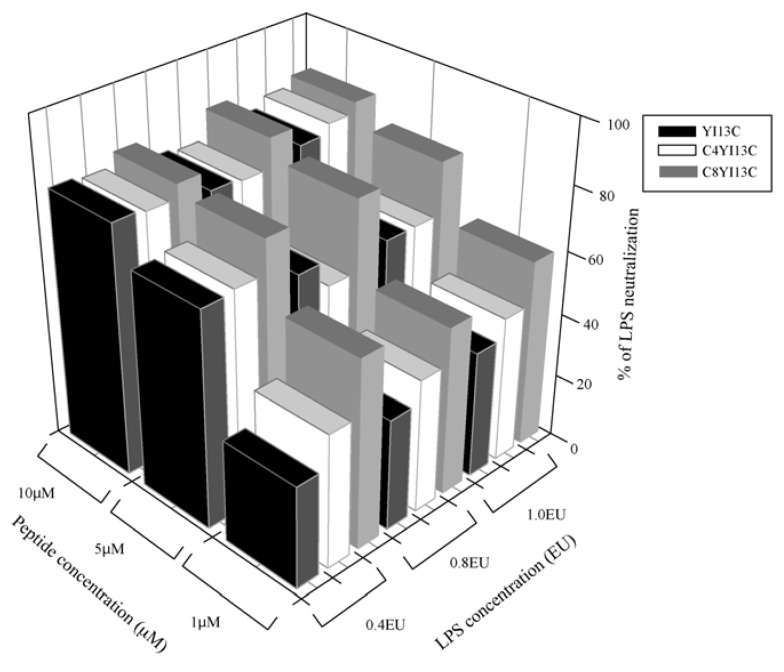
Neutralization of LPS by the designed analogs of β-boomerang peptides assayed using a limulus ameobocyte lysate assay.

In order to develop antimicrobial agents against sepsis, a compound should be active in neutralization of endotoxin. We have selected bacterially active disulfide bridged Cys analogs (YI13C, C4YI13C and C8YI13C) for LPS neutralization using chromogenic LAL kit. LAL assay was performed at three different concentrations of LPS and four different concentrations of peptides. All of the peptides tested show 80% neutralization in different concentration of LPS (expressed as endotoxin units, EU), in dose dependent fashion (Figure 2).

The non acylated YI13C peptide also shows neutralization activity equal to acylated analogs as the concentration of the peptide increases (at 10 μM peptide concentration, Figure 2), but the acylated analog C8YI13C peptide shows around 60% endotoxin neutralization of 0.4 EU even at 1.0 μM peptide concentration (Figure 2), which increases to 80% with both increases in peptide and LPS concentration. It is also noteworthy to observe that C4YI13C peptide showed ~40% LPS neutralization at a dose of 0.4 EU at 1.0 μM peptide concentration and as the concentration of LPS increases to 1.0 EU, the neutralizing activity increases to 50% (Figure 2). Therefore, it may be envisioned that the disulfide dimerization of lipopeptides renders the peptides more hydrophobic and cationic which is required for active bacterial killing and endotoxin neutralization.

### 3.4. Surface Charge Neutralization by Zeta Potential Studies

**Figure 3:**
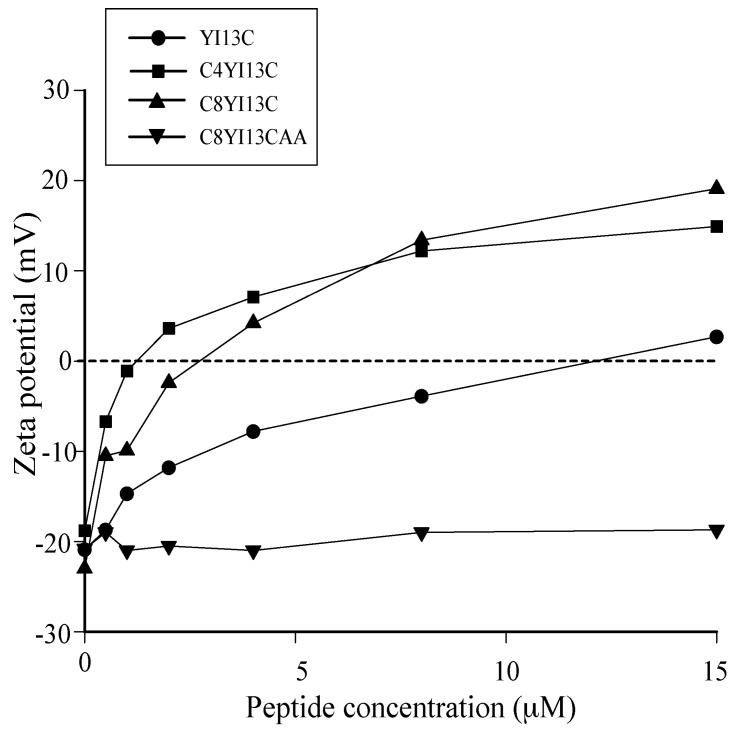
Changes of zeta potential of *E. coli* cells membranes in the presence of designed analogs of β-boomerang peptides.

Zeta potential is widely used to quantify the surface charges of bacterial cells that are readily available for the AMPs to neutralize and thereby disrupt the cells [49]. Gram-negative bacteria generally exhibit a large negative potential because of the presence of carboxylate and phosphate groups on the outer membrane surface. When *E. coli* cells were analyzed in the absence of peptides, it exhibited a negative potential of about −20.0 mV (Figure 3). Additions of increasing concentrations of active YI13C, C4YI13C and C8YI13C peptides effectively neutralize negative charge of the bacterial cell surface (Figure 3). After addition of 4 μM of C4YI13C and C8YI13C, over compensation of surface charge of about +19.1 and +24.0 was noted, respectively (Figure 3). Such an over compensation of charges were generally observed when the peptides are inserted into the hydrophobic milieu of membrane. On the other hand, the inactive C8YI13CAA did not show any detectable change in zeta potential (Figure 3). This clearly indicates that the disulfide dimerized and acylated analogs of β-boomerang peptides effectively interact with the membrane surface, neutralize the surface charge and then reach out to the hydrophobic environment of the bacterial cells plausibly leading to cell lysis.

### 3.5. Outer Membrane Permeability by NPN Assay

**Figure 4:**
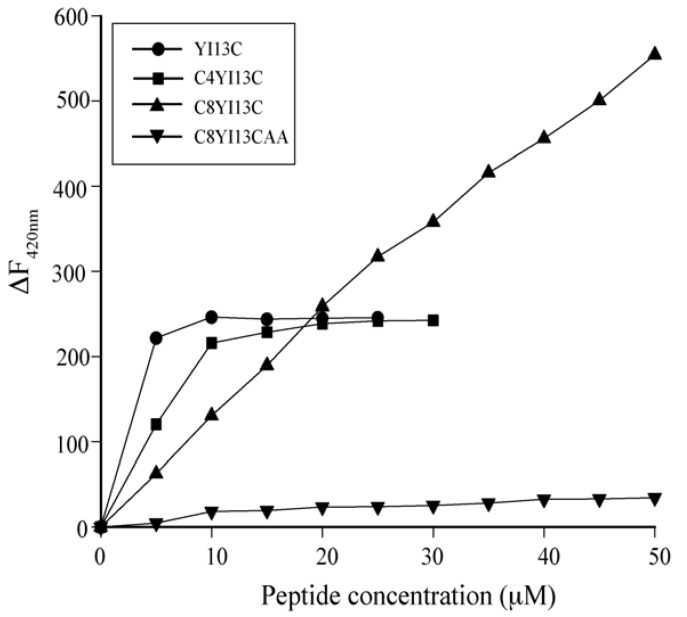
Outer membrane permeabilization induced by designed analogs of β-boomerang peptides using NPN dye. ∆F_420nm_ is the difference between fluorescence intensities after and before additions of peptides at emission maxima at 420 nm.

Designed peptides were examined for permeabilization of the outer membrane of *E. coli* cells using a hydrophobic fluorescence probe, 1-N-phenylnapthylamine (NPN). NPN cannot partition into the membrane and has weak fluorescence in aqueous environment. If the integrity of outer membrane is perturbed, then NPN can partition into hydrophobic environment and can exhibit high intensity fluorescence. The ability to permeabilize outer membrane was dose dependent *i.e*., as the concentration of peptide increases; the fluorescent intensity of NPN also increases for active peptide analogs (Figure 4). At around 10 μM of YI13C and C4YI13C, the fluorescence intensity reaches a plateau, whereas it continues to increase for C8YI13C (Figure 4). There is no marked increase in intensity for inactive C8YI13CAA peptide (Figure 4).

### 3.6. Intrinsic Tryptophan Fluorescence and Acrylamide Quenching

Tryptophan is an excellent intrinsic fluorescent probe to monitor the interactions of the peptides with lipids and detergent micelles like LPS and DPC, respectively. The intrinsic fluorescence of the free peptides showed an emission maximum around 354 nm–358 nm indicating maximum exposure of Trp residue to the aqueous environment (Table 3). The presence of blue shift *i.e*., shift of the emission maximum towards shorter wavelengths is an indication of the incorporation of the peptides into the hydrophobic environment of the micelles [45]. In the presence of negatively charged LPS lipid micelles all of the three peptides, YI13C, C4YI13C and C8YI13C, experience a higher blue shift in Trp emission wavelength in comparison to emission maxima observed in the zwitterionic DPC micelles (Table 3). Also, among the three peptides, the extent of the blue shift is noticed to be highest for C8YI13C, possibly due of the longer hydrophobic acyl chain.

**Table 3:** Intrinsic tryptophan emission maxima (λmax) and Stern-Volmer constant (K_sv_) values of Trp residue in buffer, LPS and DPC micelles.

Peptides	λmax			Stern-Volmer constant (K_sv_)		
	Free	LPS	DPC	Free	LPS	DPC
YI13C	356	339	354	43.1	6.0	11.1
C4YI13C	358	338	348	37.9	5.8	11.3
C8YI13C	356	334	344	23.3	5.0	11.2

Quenching of Trp fluorescence of the free peptide and peptide-LPS, peptide-DPC complexes with neutral quencher, acrylamide, further assessed the extent of incorporation of the fluorophore into hydrophobic environment of lipids. The exposure of Trp residue to the quencher is maximum when it is exposed to aqueous environment and minimum when it is buried inside the hydrophobic environment (Table 3). Table 3 shows the Stern-Volmer constants of the peptides in free and in complexes with LPS and DPC. As can be seen, the free peptide has a very high K_sv_ value indicating a maximum exposure of the tryptophan residue to the quencher. In the presence of LPS, the quenching constant values decreased drastically, demonstrating that the tryptophan residue is not solvent exposed and buried inside LPS micelles. On the other hand, Ksv values are two times more in DPC micelles compared to LPS, indicating that the peptides are membrane selective and the interactions with DPC micelles could be limited to the surface of the membrane. These results correlate well with the observed high bactericidal and low hemolytic activities of the designed peptides.

### 3.7. Dissociation of FITC-LPS Aggregates

**Figure 5:**
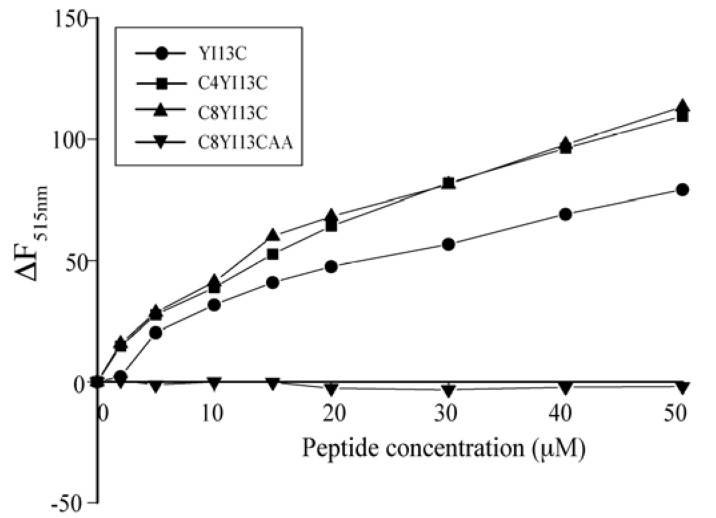
Dissociation of FITC conjugated LPS micelles by designed analogs of β-boomerang peptides. ∆F_515nm_ is the difference between fluorescence intensities after and before additions of peptides at emission maxima of FITC at 515 nm.

FITC fluorescence is highly quenched in FITC-LPS micelles due to close proximity of chromophores in LPS micelle structure. Structural perturbation and dissociation of LPS micelle aggregates alleviate quenching of FITC fluorescence. In parallel with the outer membrane permeability assay, additions of the active peptides showed concomitant increases in fluorescent intensity in a dose dependent manner (Figure 5). Acylated analogs were found to have slightly higher dissociating ability than YI13C peptide (Figure 5). The fluorescence intensity of FITC remained unchanged even when the concentrations of inactive C8YI13CAA increased (Figure 5).

### 3.8. Dynamic Light Scattering Measurements

**Figure 6:**
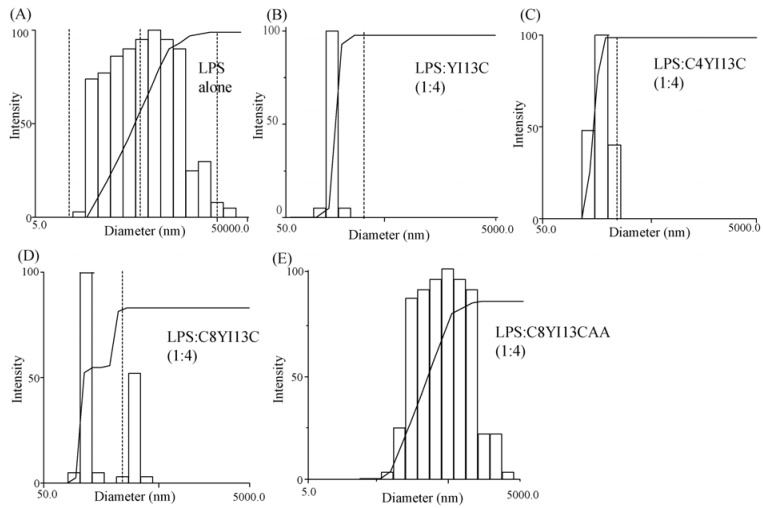
Disaggregation of LPS micelles by designed analogs of β-boomerang peptides using dynamic light scattering experiments.

To further validate the ability of the peptides to dissociate LPS aggregates, dynamic light scattering measurements were carried out. In the absence of peptides, LPS displayed a wide distribution of particles ranging from 5.0 to 50,000 nm with 814 nm sized aggregates constituting the most abundant population (Table 4, Figure 6). As expected, with the addition of increasing concentration of active peptide analogs delineated dissociation of larger aggregates of LPS into smaller size aggregates. At the ratio of LPS: peptide 1:4, only smaller aggregates of diameter 291, 331 and 385 nm were present for YI13WFC, C4WFC and C8WFC, respectively (Figure 6, Table 4). The distribution of LPS micelles also decreased from 5.0–50,000 nm to 50.0–5000 nm. The inactive C8YI13CAA peptide displayed a meager dissociation of larger aggregates of LPS with 630 nm of particles dominating (Figure 6, Table 4). DLS experiments of isolated peptides did not yield any detectable scattering due to their small sizes (data not shown).

**Table 4:** Dissociation of aggregates of LPS micelles to smaller particles by hybrid peptides.

LPS:Peptide	Diameter (nm)
LPS	814
LPS: YI13C (1:4)	291
LPS: C4YI13C (1:4)	331
LPS: C8YI13C (1:4)	385
LPS: C8YI13CAA (1:4)	630

### 3.9. Isothermal Titration Calorimetry Studies

The thermodynamic parameters that dictate LPS-peptide interactions were determined using ITC. In these experiments, peptide samples were injected into a fixed concentration of LPS placed in the cell at 25 °C. ITC thermal profiles of YI13C and C4YI13C peptides interactions with LPS demonstrated both endothermic and exothermic processes. At the beginning of the titrations, positive ITC peaks indicated endothermic binding interactions, which were followed by downward peaks signifying exothermic interactions. AMPs-LPS interactions are generally characterized by an endothermic process at 25 °C, below the phase transition temperature of LPS [50]. The endothermic binding would indicate the dominance of hydrophobic interactions in complex formation, whereas exothermic binding would imply a predominant role of polar interactions, e.g., ionic and/or hydrogen bond formation in the complex formation. The observed biphasic behavior of the binding interactions between designed peptides and LPS may occur due to an entropy gain, in the form of release of water molecules from phosphate groups of LPS, followed by strong ionic interactions between LPS and peptides. ITC data were fitted into a set of single site binding mode yielding interactions parameters (Table 5).

**Table 5:** Thermodynamic parameters and binding affinity of isoleucine and cysteine analogs with LPS micelles.

Binding Parameters	YI13C	C4WFC
K_a_ (µM^−1^)	4.2	2.2
ΔH (kcal.mol^−1)^	4.6	3.0
TΔS(kcal.mol^−1^deg^−1^)	13.6	11.6
ΔG (kcal.mol^−1^)	−9.03	−8.6
K_d_ (µM)	0.23	0.45

**Figure 7:**
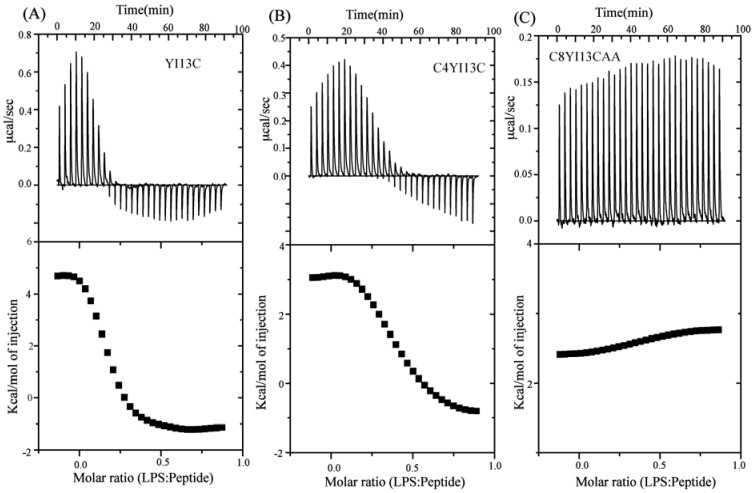
ITC thermograms of interactions between LPS and designed analogs of β-boomerang peptides.

The C8YI13CAA peptide interacted weakly with LPS as indicated by lack of saturation (Figure 7C). The equilibrium dissociation constant (K_d_) of YI13C and C4YI13C peptides estimated to be 0.23 μM and 0.45 μM, respectively (Table 5). The LPS binding affinity of these peptides appear to be higher as compared to the parent β-boomerang peptide [42].

### 3.10. Structural Characterization by NMR Spectroscopy

The solution conformations of YI13C, C4YI13C peptides were examined in free solution using two-dimensional ^1^H-^1^H NOESY and TOCSY experiments. C8YI13C and C8YI13CAA peptides showed limited solubility and have not been investigated by NMR.

**Figure 8:**
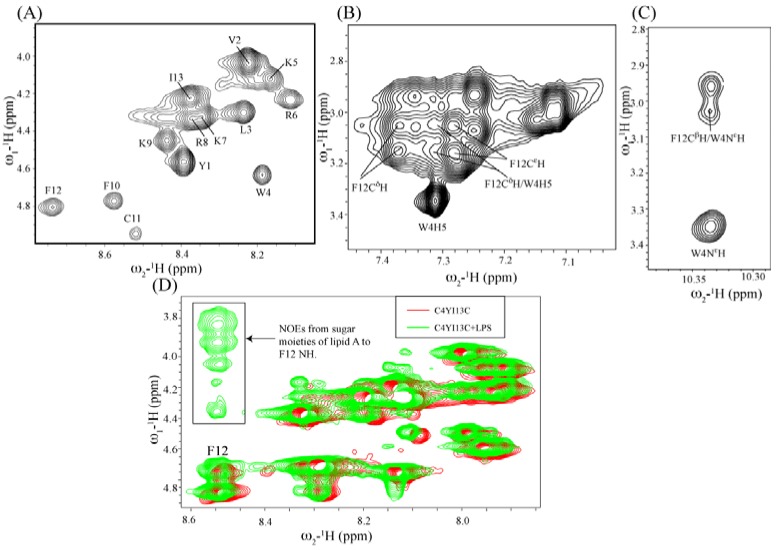
Solution NMR studies of YI13C and C4YI13C peptides by NMR spectroscopy. (**A**) Sequence specific resonance assignment in the finger print region of two dimensional ^1^H-^1^H TOCSY spectrum of C4YI13C in aqueous media at 278 K. (**B** and **C**) Long range NOEs of C4YI13C corresponding to indole ring of W4 and F12 beta proton. (**D**) Overlay of free (red) and tr-NOE (green) spectra of C4YI13C delineating signals from sugar moieties, indicated by an arrow, of LPS resonating along amide proton of F12.

1-D NMR spectra of YI13C, C4YI13C were obtained at different temperatures and pH. Samples at pH 3.5 and temperature at 278 K exhibited high quality NMR spectra. Sequence-specific resonance assignments of the peptides were achieved by combined analyses of 2-D TOCSY and NOESY spectra. Figure 8A shows the fingerprint region of the 2-D TOCSY spectrum of C4YI13C peptide. As can be seen, NH/CαH correlations are unambiguously detected for all 13 residues, including Y1. Note that the amide proton of residue Y1 of C4YI13C peptide is involved in a covalent bonding with the carboxylate of group of the acyl chain. Analyses of NOESY spectra of C4YI13C peptide revealed sequential and medium range NOEs of *i* to *i +* 1, and *i* to *i +* 2. The aromatic ring protons of residues Y1 and W4 showed NOE contacts. Interestingly, C4YI13C peptide demonstrated long range NOE connectivities between aromatic side chains of residues W4 and F12 (Figure 8B). The N^ε^H proton of the indole ring of W4 revealed NOE cross peak with the beta protons of F12 (Figure 8C). The acyl chain resonance of C4YI13C showed NOE only to residue Y1, indicating absence of packing of the acyl chain with other residues. A similar NOE pattern was observed for the non-acylated YI13C peptide, although the long-range NOEs between residues W4 and F12 were found to be of relatively low intensity (data not shown).

### 3.11. NMR Studies of YI13C and C4YI13C in LPS

Atomic-resolution structures of AMPs in complex with LPS have been determined using transferred nuclear Overhauser effect spectroscopy (tr-NOESY) [51]. In tr-NOESY, NOEs of the ligand bound to a macromolecule are transferred to the resonances of the free ligand while ligand and macromolecule interactions are under the fast exchange regime in NMR time scale [32]. A high-affinity binding interactions may not show tr-NOEs due to the slow dissociation of the complex. 1-D NMR spectra of either YI13C or C4YI13C were acquired in aqueous solutions at various concentrations of LPS. NMR spectra of both peptides demarcated LPS concentration dependent diminution of signal intensity without any observable line broadening effect, indicating a plausible slow dissociation of the peptide-LPS complex resulting from high-affinity interactions. ITC studies indeed showed that YI13C and C4YI13C peptides bind to LPS with higher affinity in comparison to the parent YI12WF peptide (Table 5). We have acquired 2-D NOESY spectra of C4YI13C peptide in presence of LPS. C4YI13C peptide showed very similar NOE connectivity as observed for free peptide, indicating the absence of tr-NOESY related cross-peaks. Interestingly, we detected inter-molecular NOEs involving resonances of the sugar moieties of lipid A with residue F12 of C4WFC peptide (Figure 8D). This observation demonstrates that the designed peptide is in close proximity <5Å to the lipid A moiety of LPS. However, due to the absence of LPS bound structure of the peptide, a reliable interface between LPS micelles and C4WFC could not be established based on the chemical shift changes and/or inter-molecular NOEs. In particular, the cationic sidechains of C4WFC were not well resolved in the 3D structure of the isolated peptide in LPS free solution.

### 3.12. Structure of C4YI13C Peptide in Aqueous Solution

**Figure 9:**
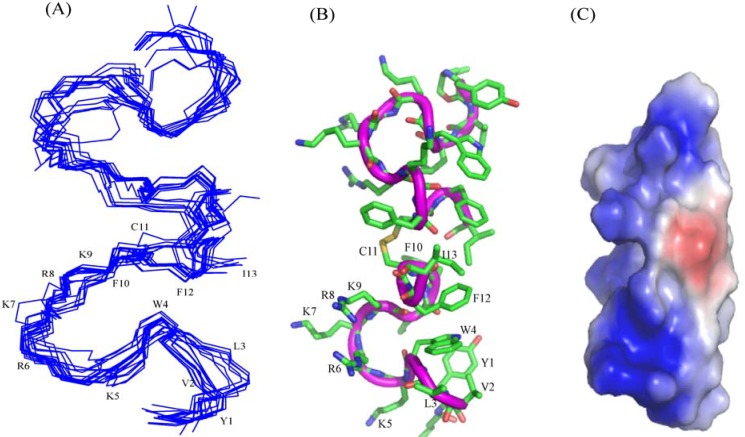
(**A**) Superimposition of backbone of 10 lowest energy structures of homo-dimeric C4YI13C in aqueous solution. (**B**) A representative structure of the homo dimeric C4YI13C showing sidechain orientation (**C**) Electrostatic surface potential of the homo dimeric C4YI13C.

An ensemble of 3-D structures of the homodimeric acylated C4YI13C peptide, linked by a disulfide bridge, has been determined based on 207 NOE driven distance constraints using CYANA (Figure 9, Table 6). The RMSD values for backbone atoms and for all the heavy atoms are confined to 0.76 Å and 1.6Å, respectively (Table 6). The 3-D structure of C4YI13C peptide is determined by two β-boomerang like structures stabilized by the disulfide bridge (Figure 9A).

**Table 6:** Summary of structural statistics of 20 lowest energy structures of dimeric C4YI13C in aqueous solution.

Distance restraints	
intraresidue (|i − j| =0)	22
sequential (|i − j| = 1)	110
medium range (2 ≤ |i − j| ≤ 4)	48
long range (|i − j| > 5)	26
total NOE constraints	207
Deviation from mean structure	
backbone atoms (N,Cα, C`) (Å)	0.76
heavy atoms (Å)	1.6
Ramachandran plot for the mean structure	
% residues in the most favourable and additionally allowed regions	100
% residues in the generously allowed region	0
% residues in the disallowed region	0

The dimeric structure of C4YI13C is amphipathic, whereby the aromatic and aliphatic sidechains of residues and sidechains of cationic residues are segregated on two different faces of the molecule (Figure 9B). The electrostatic potential of the dimer reveals a large surface of cationic charge and an extended region of non-polar surface (Figure 9C).

## 4. Conclusions

β-Boomerangs are *de novo* designed antimicrobial and antiendotoxic peptides. These peptides demonstrate LPS binding as a mode of antibacterial action and endotoxin neutralization. LPS-binding stabilizes the β-boomerang conformation of these peptides. Unique amino acid sequences, novel structures and selective interactions with negatively charged lipids of β-boomerang peptides provide an attractive template for the development of potent antibacterial and antiendotoxic molecules. Several research groups have designed AMPs, based on physicochemical properties and structural propensities including amphipathic α-helical and β-hairpins [5,6,52,53,54,55]. The β-boomerang AMPs appear to be distinct from these designed AMPs, in terms of structures and amphipathicity [41,42]. In particular, the mode of action of β-boomerang peptides includes strong perturbation of outer membrane LPS of Gram-negative bacteria. The Gram-positive bacteria contain lipoteichoic acid as a part of the cell wall structure in peptidoglycan layer. At present, interactions of β-boomerang peptides with lipoteichoic acid are yet to be determined.

In this work, we have designed and characterized analogs of β-boomerang peptides, termed YI13C, C4YI3C, C8YI13C, for broad spectrum antibacterial activity and LPS neutralization. These peptides contain a short acyl chain, C4 and C8, at their N-termini and dimerize through a disulfide bond at their C-termini. Designed peptides YI13C, C4YI3C, C8YI13C demonstrated potent bactericidal and endotoxin neutralization compared to the parent peptide. The acylated analogs are found to more active in comparison to the non-acylated peptide YI13C. These peptides are low in hemolytic activity and interacted specifically with negatively charged LPS like lipids. The high-affinity interactions of these peptides with LPS are vital for permeabilization of outer membrane, endotoxin neutralization and structural perturbation of LPS micelles. The 3-D structure of the peptide C4YI13C in free solution revealed a β-boomerang-like fold with an amphipathic disposition of the cationic and hydrophobic sidechains.

## References

[1] Zasloff M. (2002). Antimicrobial peptides of multicellular organisms. Nature.

[2] Nguyen L.T., Haney E.F., Vogel H.J. (2011). The expanding scope of antimicrobial peptide structures and their modes of action. Trends. Biotechnol..

[3] Fjell C.D., Hiss J.A., Hancock R.E.W., Schneider G. (2012). Designing antimicrobial peptides: Form follows function. Nat. Rev. Drug Discov..

[4] Ganz T., Lehrer R.I. (1998). Antimicrobial peptides of vertebrates. Curr. Opin. Immunol..

[5] Shai Y. (2002). From innate immunity to *de-novo* designed antimicrobial peptides. Curr. Pharm. Des..

[6] Tossi A., Sandri L., Giangaspero A. (2000). Amphipathic alpha helical antimicrobial peptides. Biopolym. (Pept. Sci.).

[7] Boman H.G. (1995). Peptide antibiotics and their role in innate immunity. Annu. Rev. Immunol..

[8] Brown K.L., Hancock R.E.W. (2006). Cationic host defense (antimicrobial) peptides. Curr. Opin. Immunol..

[9] Jenssen H., Powers H., Hancock R.E.W. (2006). Peptide antimicrobial agents. Clin. Microbiol. Rev..

[10] Hancock R.E.W., Lehrer R.I. (1998). Cationic peptides: A new source of antibiotics. Trends. Biotechnol..

[11] Aoki W., Ueda M. (2013). Characterisation of antimicrobial peptides toward the development of novel antibiotics. Pharmaceuticals.

[12] Brogden N.K., Brogden K.A. (2011). Will new generations of modified antimicrobial peptides improve their potential as pharmaceuticals?. Int. J. Antimicrob. Agents.

[13] Epand R.M., Vogel H.J. (1999). Diversity of antimicrobial peptides and their mechanisms of action. Biochim. Biophys. Acta.

[14] Bhunia A., Saravanan R., Mohanram H., Mangoni M.L., Bhattacharjya S. (2011). NMR structures and interactions of temporin-1Tl and temporin-1Tb with lipopolysaccharide micelles. Mechanistic insights into outer membrane permeabilization and synergisitc activity. J. Biol. Chem..

[15] Mohanram H., Bhattacharjya S. (2014). Resurrecting inactive antimicrobial peptides from the lipopolysaccharide trap. Antimicrob. Agents Chemother..

[16] Barnickel G., Bradaczek H., Naumann D., Rietschel E.T., Giesbrecht P., Labischinski H. (1985). High state of order of isolated bacterial lipopolysaccharide and its possible contribution to the permeation barrier property of the outer membrane. J. Bacteriol..

[17] Allende D., McIntosh T.J. (2003). Lipopolysaccharides in bacterial membranes act like cholesterol in eukaryotic plasma membranes in providing protection against melittin-induced bilayer lysis. Biochemistry.

[18] Mangoni M.L., Epand R.F., Rosenfeld Y., Peleg A., Barra D., Epand R.M., Shai Y. (2008). Lipopolysaccharide, a key molecule involved in the synergism between temporins in inhibiting bacterial growth and in endotoxin neutralization. J. Biol. Chem..

[19] Marra M.N., Wilde C.G., Griffith J.E., Snable J.L., Scott R.W. (1990). Bactericidal/permeability increasing protein has endotoxin neutralizing ability. J. Immunol..

[20] Bhattacharjya S. (2010). De novo Designed lipopolysaccharide binding peptides: structure based development of antiendotoxic and antimicrobial drugs. Curr. Med. Chem..

[21] Raetz C.R., Whitfield C. (2002). Lipopolysaccharide endotoxins. Annu. Rev. Biochem..

[22] Xu J., Kochanek K.D., Tejada-vera B. (2009). Deaths: Preliminary data for 2007. National Vital Statistics Report.

[23] David S.A. (2001). Towards a rational development of anti-endotoxin agents: Novel approaches to sequestration of bacterial endotoxins with small molecules. J. Mol. Recognit..

[24] Bowdish D.M.E., Hancock R.E.W. (2005). Anti-endotoxin properties of cationic host defence peptides and proteins. J. Endotoxin. Res..

[25] Lehrer R.I., Lichtenstein A.K., Ganz T. (1993). Defensins: antimicrobial and cytotoxic peptides of mammlian cells. Annu. Rev. Immunol..

[26] Kokryakov V.N., Harwig S.S.L., Panyutich E.A., Shevchenko A.A., Aleshina G.M., Shamova O.V., Korneva H.A., Lehrer R.I. (1993). Protegrins: Leukocyte antimicrobial peptides that combine features of corticostatic defensins and tachyplesins. FEBS.

[27] Matsuzaki K. (1999). Why and how are peptide-lipid interactions utilized for self defense? Magainins and tachyplesins as archetypes. Biochim. Biophys. Acta.

[28] Harwig S.S.L., Waring A., Yang H.J., Cho Y., Tan L., Lehrer R.I. (1996). Intramolecular disulfide bonds enhance the antimicrobial and lytic activities of protegrins at physiological sodium chloride concentrations. Eur. J. Biochem..

[29] Mani R., Waring A.J., Lehrer R.I., Hong M. (2005). Membrane disruptive abilities of beta hairpin antimicrobial peptides correlate with conformation and activity: A 31P and 1H NMR study. Biochim. Biophys. Acta.

[30] Varkey J., Nagaraj R. (2005). Antibacterial activity of human neutrophil defensin HNP-1 analogs without cysteines. Antimicrob. Agents Chemother..

[31] Haney E.F., Vogel H.J. (2009). NMR of antimicrobial peptides. Annu. Rep. NMR.

[32] Findlay B., Zhanel G.G., Schweizer F. (2010). Cationic amphiphiles, a new generation of antimicrobials inspired by the natural antimicrobial peptide scaffold. Antimicrob. Agents Chemother..

[33] Andra J., Lohner K., Blondelle S.E., Jerala R., Moriyon I., Koch M.H., Garidel P., Brandenburg K. (2005). Enhancement of endotoxin neutralization by coupling of a C12-alkyl chain to a lactoferricin derived peptide. Biochem. J..

[34] Mangoni M.L., Shai Y. (2011). Short native antimicrobial peptides and engineered ultrashort lipopeptides: Similarities and differences in cell specificities and modes of action. Cell. Mol. Life Sci..

[35] Tsubery H., Ofek I., Cohen S., Fridkin M. (2001). N-terminal modifications of Polymyxin B nonapeptide and their effect on antibacterial activity. Peptides.

[36] Farnaud S., Evans R.W. (2003). Lactoferrin-a multifunctional protein with antimicrobial properties. Mol. Immunol..

[37] Majerle A., Kidric J., Jerala R. (2003). Enhancement of antibacterial and lipopolysacchride binding activities of a human lactoferrin peptide fragment by the addition of acyl chain. J. Antimicrob. Chemother..

[38] Rosenfeld Y., Lev N., Shai Y. (2010). Effect of the hydrophobicity to net positive charge ratio on antibacterial and anti-endotoxin activities of structurally similar antimicrobial peptides. Biochemistry.

[39] Lockwood N.A., Haseman J.R., Tirrell M.V., Mayo K.H. (2004). Acylation of SC4 dodecapeptide increases bactericidal potency against Gram positive bacteria, including drug resistant strains. Biochem. J..

[40] Etzerodt T., Henriksen J.R., Rasmussen P., Clausen M.H., Andresen T.L. (2011). Selective acylation enhances membrane charge sensitivity of the antimicrobial peptide Mastoporan-X. Biophys. J..

[41] Bhattacharjya S., Domadia P.N., Bhunia A., Malladi S., David S.A. (2007). High-resolution solution structure of a designed peptide bound to lipopolysaccharide: Transferred nuclear Overhauser effects, micelle selectivity, and anti-endotoxic activity. Biochemistry.

[42] Bhunia A., Mohanram H., Domadia P.N., Torres J., Bhattacharjya S. (2009). Designed beta-boomerang antiendotoxic and antimicrobial peptides: sturctures and activities in lipopolysaccharide. J. Bio. Chem..

[43] Bhunia A., Geoklin C., Domadia P.N., Warshakoon H., Cromer J.R., David S.A., Bhattacharjya S. (2008). Interactions of a designed peptide with lipopolysaccharide: Bound conformation and anti-endotoxic activity. Biochem. Biophys. Res. Commun..

[44] Bhunia A., Mohanram H., Bhattacharjya S. (2008). Lipopolysacchride bound structures of the active fragments of fowlicidin-1, a cathelicidin family of antimicrobial and antiendotoxic peptide from chicken, determined by transferred nuclear Overhauser effect spectroscopy. Biopolym. (Pept. Sci.).

[45] Bhunia A., Domadia P.N., Bhattacharjya S. (2007). Structural and thermodynamic analyses of the interaction between melittin and lipopolysaccharide. Biochim. Biophys. Acta.

[46] Bhattacharjya S., Ramamoorthy A. (2009). Multifunctional host defense peptides: Functional and mechanistic insights from NMR structures of potent antimicrobial peptides. FEBS J..

[47] Guntert P. (2004). Automated NMR protein structure calculation with CYANA. Meth. Mol. Biol..

[48] Laskowski R.A., Rullmann J.A.C., MacAruthur M.W., Kaptein R., Thornton J.M. (1996). AQUA and PROCHECK-NMR: Programs for checking the quality of protein structures solved by NMR. J. Biomol. NMR.

[49] Domingues M.M., Castanho M.A.R.B., Santos N.C. (2009). rBPI21 promotes lipopolysaccharide aggregation and exerts its antimicrobial effects by (hemi)fusion of PG-containing membranes. PLoS One.

[50] Srimal S., Surolia N., Balasubramanian S., Surolia A. (1996). Titration calorimetric studies to elucidate the specificity of the interactions of polymyxin B with lipopolysaccharides and lipidA. Biochem. J..

[51] Bhattacharjya S., David S.A., Mathan V.I., Balaram P. (1997). Polymyxin B nonapeptide: Conformations in water and in the lipopolysaccharide bound state determined by two-dimensional NMR and molecular dynamics. Biopolymers.

[52] Bahar A.A., Ren D. (2013). Antimicrobial peptides. Pharmaceuticals.

[53] Jiang Z., Vasil A.I., Vasil M.L., Hodges R.S. (2014). “Specificity determinants” improve therapeutic indices of two antimicrobial peptides piscidin 1 and dermaseptin S4 against the gram-negative pathogens *Acinetobacter baumannii* and *Pseudomonas aeruginosa*. Pharmaceuticals.

[54] Loose C., Jensen K., Rigoutsos I., Stephanopoulos G. (2006). A linguistic model for the rational design of antimicrobial peptides. Nature.

[55] Wang G. (2013). Database-Guided Discovery of Potent Peptides to Combat HIV-1 or Superbugs. Pharmaceuticals (Basel).

